# Attention-Deficit Hyperactivity Disorder Symptoms in Adults Diagnosed with Multiple Sclerosis: Prevalence and Correlates

**DOI:** 10.3390/jcm13133844

**Published:** 2024-06-29

**Authors:** Mariam Al-Ameri, Hanan Abu-Shaikh, Mohammad Mansour, Suha Al-Habahbeh, Feras Weshah, Wail Ennab, Ammena Y. Binsaleh, Sireen Abdul Rahim Shilbayeh, Omar Gammoh

**Affiliations:** 1 Department of Clinical Pharmacy and Pharmacy Practice, Faculty of Pharmacy, Yarmouk University, Irbid 21163, Jordan; m.alameri@yu.edu.jo; 2 Prince Hamza Hospital, Amman 11123, Jordan; hananabushaikh@gmail.com; 3 Department of Neurology, Al-Bashir Hospital, Amman 11151, Jordan; mohammadmansour981@gmail.com (M.M.); habahbehsuha@gmail.com (S.A.-H.); weshah0780475839@gmail.com (F.W.); wailennab@gmail.com (W.E.); 4 Department of Pharmacy Practice, College of Pharmacy, Princess Nourah bint Abdulrahman University, P.O. Box 84428, Riyadh 11671, Saudi Arabia; aysaleh@pnu.edu.sa (A.Y.B.); ssabdulrahim@pnu.edu.sa (S.A.R.S.)

**Keywords:** ADHD, multiple sclerosis, risk

## Abstract

**Background**: The relationship between adult ADHD symptoms in People with Multiple Sclerosis (PwMS) is understudied. This study aimed to answer two questions: are PwMS more likely to experience higher ADHD symptoms versus healthy subjects? And what are the correlates of severe ADHD symptoms in PwMS? **Methods**: This study followed a cross-sectional design with predefined inclusion criteria. The Adult ADHD Self-Report Scale-V1.1 (ASRS) was used to assess the ADHD symptoms severity. **Results**: Data were analyzed from 171 PwMS and 200 controls. Regression analysis revealed that PwMS were at a significantly (B = 3.05, t = 2.24, 95% CI = 0.37–5.73, *p* = 0.02) higher risk to report higher ADHD scores versus controls. In addition, PwMS with relapses in the last 6 months and PwMS reporting smartphone addiction were at a significantly higher risk for severe ADHD (B = 7.19, t = 269, 95% CI = 1.91–12.48, *p* = 0.008) and (B = 9.18, t = 3.47, 95% CI = 3.97–14.41, *p* = 0.001), respectively. In conclusion, diagnosis with MS in our study was identified as a risk for higher ADHD symptoms. **Conclusions**: Further research is required to establish this relationship, and holistic medical and psychological interventions are required to improve the cognitive status of PwMS.

## 1. Introduction

Attention-deficit hyperactivity disorder (ADHD) is a neurodevelopmental disorder that features hyperactivity, lack of attention, high impulsiveness, and cognitive dysfunction; taken all together, these symptoms affect the daily functioning of the affected patients, who are mainly children [1]. Unstable relationships, poor achievement at work or school, low self-esteem, and other issues can result from ADHD [2]. Even though ADHD is frequently linked to childhood, it can still exist in adults and cause particular difficulties and impairments in social, professional, and academic settings [3], as well as a markedly higher chance of developing numerous medical disorders, or worsening their severity [4]. Although ADHD is a childhood disorder affecting approximately 5% of children worldwide [5], ADHD continues in adulthood with a prevalence rate reaching up to 3% of the adult population [5,6]. The cross-talk between neuropsychiatric disorders and autoimmune diseases has been established; however, the exact association between ADHD and multiple sclerosis is poorly studied and results are still inconclusive [7]. Multiple sclerosis is an autoimmune progressive and disabling disease affecting young adults [8]. Accumulating evidence underscored the poor cognitive status among People with Multiple Sclerosis (PwMS). For example, one study that recruited about 200 PwMS reported a prevalence of cognitive dysfunction to be 34% [9]. Furthermore, other reports estimated that up to 65% of PwMS could experience cognitive dysfunctions and other debilitating symptoms including memory, attention, and information processing speed. This could be related to changes in the white matter lesions and other unknown disease-related conditions [10]. Although cognition has been studied in PwMS, adult ADHD symptoms in PwMS are still poorly studied. The symptomatology of ADHD covers several aspects including cognitive deficits. Therefore, the study of ADHD symptoms in PwMS could provide more holistic insight into the other cognitive-related symptoms experienced by PwMS.

One study that compared 72 PwMS with healthy peers showed that PwMS reported a higher rate of ADHD symptoms—36% compared to 4%—in the control group according to the Adult Attention Deficit/Hyperactivity Disorder Self-Report Scale (ASRS) [11]. In the same study, ADHD symptoms were related to the mental health burden, namely anxiety and depression. According to our knowledge, very few studies have examined whether PwMS could be at higher risk for severe ADHD symptoms, and very few studies identified potential risk factors for severe ADHD symptoms in PwMS. Therefore, the present study sought to (1) investigate whether PwMS are at higher risk for severe ADHD versus healthy controls and (2) identify the correlates of severe ADHD among PwMS.

## 2. Materials and Methods

### 2.1. Study Design and Settings

We employed a cross-sectional design with predetermined inclusion criteria. PwMS were recruited from the MS unit located in Al Bashir Hospital-Jordan. All the potential PwMS were approached upon their frequent visit to the unit where the study objective and protocol were fully explained before enrollment. The control group was randomly recruited from healthy individuals visiting the hospital with their family members to provide them assistance. A link leading to the study questionnaire was sent to all the willing participants, who were asked to fill out the written consent form electronically before enrollment. The sample size was informed by previous studies. All the participants had the right to exit from the study at any time. The study obtained ethical approval from the Yarmouk University IRB Committee (692).

### 2.2. Inclusion Criteria

PwMS with a diagnosis of RRMS based on the 2017 McDonald criteria [12] and receiving disease-modifying therapy for at least one year were recruited for this study.

### 2.3. Exclusion Criteria

PwMS diagnosed with other types of MS, those not adhering to their disease-modifying therapy for at least one year, and those newly diagnosed as PwMS were excluded from the study.

### 2.4. Study Instrument

A well-designed study instrument has been created to cover the demographical and clinical information of the study population. The demographics covered were sex, age, marital status, employment status, and smoking status. The clinical information for PwMS covered the disease duration, the disease-modifying therapy received, and the history of relapse(s) in the last six months. In addition, the addiction to smartphones for PwMS was assessed using the Smartphone Addiction Scale. This Arabic-translated validated and reliable scale (Cronbach alpha = 0.94) [13] comprises 10 questions, each one rated against a 6-point Likert scale with responses that range from 1 for “strongly disagree” up to 6 for “strongly agree”. The scale generates a score between 0 and 60 with a cut-off score of 39 and above to identify addiction to smartphones [14,15].

### 2.5. Outcome Measure

#### ADHD Symptoms

The Arabic version of the Adult ADHD Self-Report Scale-V1.1 (ASRS) was employed to evaluate the ADHD symptoms. The scale shows excellent internal consistency with a Cronbach alpha of 0.94 [16]. This scale consists of 18 items aligned with the diagnostic criteria of the Diagnostic and Statistical Manual of Mental Disorders (DSM) and generates a maximum score of 72, with a higher score indicating a higher severity of ADHD [17,18,19].

### 2.6. Data Analysis

Before the data analysis step, all the participants with incomplete data were excluded to improve the data quality and to minimize any potential bias.

The categorical variables of the demographics of the enrolled participants and the clinical information of the PwMS were described using frequencies and percentages. The continuous variables (age and ADHD scores) were described through mean ± standard deviation. The variables between the two groups were compared using the Chi-square analysis for the categorical variables and the independent t-student test for the continuous variables. To investigate whether diagnosis with MS could be a risk factor for higher ADHD scores, a univariate linear regression analysis was performed with ADHD as the dependent variable. To identify the correlates for severe ADHD symptoms in PwMS, a univariate linear regression analysis was carried out to identify potential confounders showing *p* < 0.1, and then these factors were used to feed the multivariable linear regression model built using the backward step-wise approach. Confidence intervals were set at 95% and significance at *p* < 0.05. Data were analyzed using SPSS software version 21.

## 3. Results

### 3.1. Study Sample Features

Data analysis comprised a cohort of 371 participants, of which 171 were PwMS and 200 were demographically matched controls. In regards to the PwMS group, 125 (73.1%) were females, 103 (60.2%) were married, 107 (62.6%) were unemployed, 92 (53.8%) were non-smokers, 30 (17%) reported relapses for the past 6 months, 98 (57.3%) had been diagnosed with RRMS for less than 5 years, 32 (18.6%) reported addiction to smartphones, 90 (52.6%) were on fingolimod, 47 (27.5%) on interferons, and 34 (19.9%) on dimethyl fumarate (Table 1).

### 3.2. PwMS and Risk for ADHD Severity

The mean score of ADHD was compared between the two groups through the independent t-test. The PwMS reported a significantly higher score (Mean ± SD) (30.94 ± 13.85) compared to (27.89 ± 12.42) in the control group, (t (369) = −2.23, *p* = 0.02). Moreover, according to the linear regression univariate model for ADHD as the dependent variable, PwMS were at a significantly higher risk for higher ADHD scores (B = 3.05, t = 2.24, 95% CI = 0.37–5.73, *p* = 0.02), as shown in Table 1.

### 3.3. Correlates of ADHD Symptoms in PwMS

To investigate which factors were independently associated with higher ADHD scores in the PwMS group, an initial univariate linear regression analysis was carried out followed by a multivariable linear regression model. This revealed that PwMS with “Relapses in the last 6 months” and PwMS reporting “Smartphone addiction” were at a significantly higher risk for severe ADHD (B = 7.19, t = 269, 95% CI = 1.91–12.48, *p* = 0.008) and (B = 9.18, t = 3.47, 95% CI = 3.97–14.41, *p* = 0.001), respectively, as shown in Table 2.

## 4. Discussion

The present study had two objectives: first, to examine whether PwMS are at higher risk for severe ADHD versus healthy controls, and second, to identify the correlates of severe ADHD among PwMS. Our findings reported that PwMS are at higher risk, i.e., three-fold higher risk to report severe ADHDH symptoms versus their healthy peers. Also, we report that PwMS who had previous relapses and who reported addiction to smartphone use were at higher risk for severe ADHD symptoms.

Our findings identified MS as a significant correlate for severe ADHD symptoms. In the present study, all PwMS were diagnosed with RRMS. The previous literature identified higher ADHD rates in MS types; for example, almost 45% of PwMS with RRMS type suffered ADHD [20]. Although this study identified a significant association between PwMS and ADHD symptoms, the author reinforces that this preliminary research does not claim a causal relationship between PwMS and ADHD symptoms.

Although the exact mechanism underlying this comorbidity is yet to be clarified, this could be attributed to the neurological changes seen in the grey and white matter of PwMS, the distribution of the cortical and the juxtacortical lesions, ventricles enlargement, and corpus callosum atrophy, which are all thought to correlate to cognitive dysfunction [21,22,23]. This could explain that, in our study, PwMS who reported one or more relapses were at higher risk for severe ADHD; this suggests that ADHD symptoms could deteriorate with the disease course. This is supported by a study that confirmed that three out of four PwMS who reported poor cognition at baseline continued to suffer from cognition deterioration [24]. Findings from the current study revealed that PwMS participants who had previous relapses and who reported addiction to smartphone use were at higher risk for severe ADHD symptoms, which is consistent with the existing literature. A recent systematic review made use of recent empirical data to show the relationship between adult health outcomes and smartphone addiction. Results have consistently shown an association between smartphone addiction and symptoms of both physical and mental health, such as anxiety, depression, musculoskeletal pain, and insomnia [25]. Factors such as personality features, impulsivity, and mental health issues are implicated [26,27].

ADHD, in particular, in the present study has been connected to internet and smartphone addiction, and this finding has been confirmed in several previous studies [14,28,29]. Numerous studies examining the relationship between ADHD and addiction indicate that the disorder appears to be associated with behavioral addictions [30,31]. There is strong evidence that problematic internet use addiction is linked to cognitive deficits in working memory, motor inhibitory control, attention-focused inhibition, and decision-making, as shown by a recent meta-analysis [32].

Although little research has been found to relate ADHD and smartphone use, we suggest that because PwMS are young compared to other neurological disorders, these subjects are highly and easily attracted to the continuously updating technologies presented via smartphones. The prevalence of addiction to smartphones, according to the published studies, could reach up to 26% in young populations depending on the scale and the cut-off points used [33]. The relationship between addiction to smartphones and ADHD symptoms is believed to be bi-directional, multifactorial, and complicated. For instance, individuals with problems in assurance-seeking and avoidance behaviors are prone to be addicted to their smartphones [34]; in addition, people reporting impulsivity, focusing problems, and lack of new stimuli as ADHD symptoms could be more vulnerable to smartphone addiction [35]. On the other hand, excessive smartphone use can aggravate symptoms of ADHD by lowering attention span, raising impulsivity, and interfering with daily schedules [36]. Furthermore, the overuse of smartphones reinforces the feeling of controlling the target, as well as the freedom of self-expression on social media platforms that yields rewarding feelings for people with ADHD [37,38]. In addition, many studies found that addiction to smartphones/internet in subjects with ADHD symptoms was tightly related to depression and anxiety symptoms [39].

This pioneering study provides a significant contribution to the little existing literature about ADHD symptoms in PwMS. Altogether, the novelty, the validated ADHD and smartphone addiction scales, and the robust analysis of data are strengths of this study. Conversely, this study has some limitations. For instance, the ADHD status was not monitored at baseline, i.e., at the moment of diagnosis with MS; in addition, the smartphone addiction variable was only examined in PwMS and not for the whole cohort. Furthermore, the nature of the data collected from the scales could lead to possible bias, which was managed by excluding incomplete questionnaires and using validated and reliable scales. In addition, the study design and settings did not allow for the accurate and definitive ADHD diagnosis among PwMS due to social, cultural, and other related personal barriers such as stigma to mental health in developing countries such as Jordan.

## 5. Conclusions

This study concludes that PwMS reported higher ADHD symptoms severity compared to the healthy group. PwMS with relapses and smartphone addiction were at higher risk for ADHD. Although these findings could not be recognized as confirmatory, these findings underscore the importance of implementing more thorough, large-scale, and follow-up studies to further reveal the association between MS, smartphone addiction, and ADHD symptoms in adults. Furthermore, the findings of this study open new avenues for clinicians to provide comprehensive medical and psychological care to prevent the deterioration of these disabling symptoms in this fragile population. More in-depth studies are required to fully unravel the association between MS and ADHD.

## Figures and Tables

**Table 1:** The demographics, clinical information, and ADHD scores of the two study groups.

Factor		Controls (n = 200)	PwMS (n = 171)	*p*-Value
Age (M ± SD)		37.19 ± 16.6	37.85 ± 9.83	0.67
ADHD Score (M ± SD)		27.89 ± 12.42	30.94±13.85	0.02 *
Sex	Males	65 (32.5%)	46 (26.9%)	0.25
	Females	135 (67.5%)	125 (73.1%)	
Marital Status	Single	86 (43%)	68 (39.8%)	0.59
	Married	114 (57%)	103 (60.2%)	
Employment	Unemployed	125 (62.5%)	107 (62.6%)	0.99
	Employed	75 (37.5%)	64 (37.4%)	
Smoking Status	Non-smoker	125 (62.5%)	92 (53.8%)	0.10
	Smoker	75 (37.5%)	79 (46.2%)	
MS Relapses during the last 6 Months	No	N/A	141 (82.5%)	
	Yes	N/A	30 (17.5%)	
MS Duration	For less than 5 years	N/A	98 (57.3%)	
	For 5 years or more	N/A	73 (42.7%)	
Addiction to Smartphones	No	N/A	140 (81.4%)	
	Yes	N/A	32 (18.6%)	
Fingolimod		N/A	90 (52.6%)	
Dimethyl fumarate		N/A	34 (19.9%)	
Interferons		N/A	47 (27.5%)	

The independent t-student test was performed to compare means of the continuous variables “age” and “ADHD” scores. The other categorical variables were compared using Chi-square analysis. ADHD: attention-deficit hyperactivity disorder according to the ASRS scale; MS: multiple sclerosis, N/A: not applicable M: mean, SD: standard deviation, * *p* < 0.05.

**Table 2:** The association between PwMS covariates and ADHD severity using univariate and multivariable linear regression models for ADHD (dependent variable) according to the ASRS scale.

Factor	Univariate Analysis				Multivariable Analysis			
	B	t	95% CI	*p*	B	t	95% CI	*p*
Increasing age	0.05	0.54	−0.15–0.27	0.54	---	---	---	---
Female	1.95	0.81	−2.77–6.67	0.42	---	---	---	---
Married	1.28	0.59	−3.01–5.36	0.55	---	---	---	---
Employed	−3.26	−149	−7.57–1.04	0.14	---	---	---	---
Smoker	−0.54	−0.25	−4.74–6.67	0.80	---	---	---	---
MS duration	1.91	0.89	−2.32–6.14	0.38	---	---	---	---
Relapses in the last 6 months	6.29	2.29	0.86–11.72	0.02 *	7.19	2.69	1.91–12.48	0.008 *
Smartphone addiction	8.49	3.17	3.20–13.79	0.002 *	9.18	3.47	3.97–14.41	0.001 *
Fingolimod	−0.17	−0.08	−4.37–4.03	0.94	---	---	---	---
Dimethyl fumarate	−0.23	−0.08	−5.49–5.02	0.93	---	---	---	---
Interferons	0.39	0.16	−4.30–5.09	0.87	---	---	---	---

MS: multiple sclerosis, B: beta coefficient, t: t value, CI: confidence interval, * *p* < 0.05, r^2^ = 0.095.

## Data Availability

Data will be available from the corresponding author upon request.
